# Supplementary material for: The genome sequence of the Black Hairstreak, Satyrium pruni (Linnaeus, 1758) (Lepidoptera: Lycaenidae)
Source: Wellcome Open Res. 2025 Jul 28;10:377. [Version 1] doi: 10.12688/wellcomeopenres.24619.1 (PMC12417987; doi:10.12688/wellcomeopenres.24619.1)
Supplement: Peer review history

# Reviewer report: Reviewer response for version 1

**DOI:** 10.21956/wellcomeopenres.27129.r129025

**Published:** 2025-09-08

**Authors:** Christopher J. Fields

**Affiliations:** 1 Carver Biotechnology Center, University of Illinois Urbana-Champaign, Urbana, 61801, USA

The report for the Black Hairstreak genome is quite comprehensive and is fairly easy to follow. I also particularly like the application of chromosome painting in this particular report, which is a nice unique analysis for this particular species adding some useful additional biological context. Also happy to see that haplotype-phased assembly was pursued; past contributions seemed to focus on only the partially phased primary assembly.

 The only small concern is from some confusion regarding specifically which assembly was used for HiC scaffolding (or whether both haplotype assemblies were used).  In this section (pg 4, under ‘Genome assembly’):

*The HiFi reads were assembled using Hifiasm in Hi-C phasing mode (Chenget al., 2021; Cheng et al., 2022), producing two haplotypes. Hi-C reads (Rao et al., 2014) were mapped to the primary contigs using bwa-mem2 (Vasimuddin et al., 2019). Contigs were further scaffolded with Hi-C data in YaHS (Zhou et al., 2023), using the --break option for handling potential misassemblies. The scaffolded assemblies were evaluated using Gfastats (Formenti et al., 2022), BUSCO (Manni et al., 2021) and MERQURY.FK (Rhie et al., 2020*

 In the above and in the full results it’s pretty obvious two haplotypes were generated as the final products, but the confusing part is this: ‘Hi-C reads (Rao et al., 2014) were mapped to the **primary contigs** using bwa-mem2 (Vasimuddin et al., 2019).’  To me this would mean the pre-phased assembly, the 'p_ctg' file from hifiasm, which I have seen reported in other Wellcome papers. I’m assuming this is supposed to be hap1?  Or were HiC reads aligned to both and filtered (ala HapHiC) to scaffold both? Would be great to clarify this.

Are sufficient details of methods and materials provided to allow replication by others?

Yes

Is the rationale for creating the dataset(s) clearly described?

Yes

Are the datasets clearly presented in a useable and accessible format?

Yes

Are the protocols appropriate and is the work technically sound?

Yes

Reviewer Expertise:

Computational genomics

I confirm that I have read this submission and believe that I have an appropriate level of expertise to confirm that it is of an acceptable scientific standard.

# Reviewer report: Reviewer response for version 1

**DOI:** 10.21956/wellcomeopenres.27129.r131172

**Published:** 2025-09-04

**Authors:** Frederik Hendrickx

**Affiliations:** 1 Royal Belgian institute of natural Sciences, Brussels, Belgium

This manuscript describes the genome sequence of the Black Hairstreak ( *Satyrium pruni)*, a widespread though endangered butterfly from Europe. The genome, assembled from a single male using both PacBio HiFi and Hi-C data is of very high quality and contiguity. Based on the Hi-C contact map, 99% of the total contig length nicely assembles into 23 pseudomolecules, illustrating that the assembly is of very high quality. The homology with the Merian elements adds additional information of the quality and correctness of the assembly. As a minor point, I would suggest that the authors briefly explain what Merian elements are as it is a rather specialized term that is only used in butterfly genomics. At least reference to a paper that explains this term should be added. I have no further suggestions for improvement.

Are sufficient details of methods and materials provided to allow replication by others?

Yes

Is the rationale for creating the dataset(s) clearly described?

Yes

Are the datasets clearly presented in a useable and accessible format?

Yes

Are the protocols appropriate and is the work technically sound?

Yes

Reviewer Expertise:

Insect evolutionary genomics

I confirm that I have read this submission and believe that I have an appropriate level of expertise to confirm that it is of an acceptable scientific standard.
